# Phytate Degradation by Fungi and Bacteria that Inhabit Sawdust and Coffee Residue Composts

**DOI:** 10.1264/jsme2.ME12083

**Published:** 2012-10-26

**Authors:** Mohamed Fathallh Eida, Toshinori Nagaoka, Jun Wasaki, Kenji Kouno

**Affiliations:** 1 Graduate School of Biosphere Science, Hiroshima University, 1–4–4, Kagamiyama, Higashi-hiroshima, Hiroshima 739–8528, Japan; 2 Agricultural Microbiology Department, National Research Center, 33 El Behous St., Dokki, 12622 Cairo, Egypt

**Keywords:** phytate, degradation, bacteria, fungi, compost

## Abstract

Phytate is the primary source of organic phosphorus, but it cannot be directly utilized by plants and is strongly adsorbed by the soil, reducing bioavailability. Composting is a process used to improve the bioavailability of phytate in organic wastes through degradation by microorganisms. In this study, we aimed to investigate the phytate-degrading ability of fungi and bacteria that inhabit sawdust compost and coffee residue compost, and their contribution to the composting process. In the plate assay, the fungi that formed clear zones around their colonies belonged to the genera *Mucor*, *Penicillium*, *Galactomyces*, *Coniochaeta*, *Aspergillus*, and *Fusarium*, while the bacteria belonged to the genera *Pseudomonas*, *Enterobacter*, *Chitinophaga*, and *Rahnella*. Eight fungal isolates (genera *Mucor*, *Penicillium*, *Galactomyces*, and *Coniochaeta*) and four bacterial isolates (genera *Pseudomonas*, *Enterobacter*, and *Rahnella*) were selected to evaluate phytase activity in their liquid culture and their ability to degrade phytate in organic materials composed of mushroom media residue and rice bran. The selected fungi degraded phytate in organic materials to varying degrees. *Penicillium* isolates showed the highest degradation ability and *Coniochaeta* isolate exhibited relatively high degradation ability. The clear zone diameters of these fungal isolates displayed significantly positive and negative correlations with inorganic and phytate phosphorus contents in the organic materials after incubation, respectively; however, none of the selected bacteria reduced phytate phosphorus in organic materials. It is therefore possible that fungi are major contributors to phytate degradation during composting.

Composting is the process used to transform organic wastes accumulated from human activity and other waste biomass into substances that may be used for fertilizing the soil and improving the growth of cultivated plants (33). The degradation processes during composting are mainly catalyzed by the action of microorganisms (58). To assess the composting process, it is important to study and understand the roles of microbial communities in the degradation processes. As a result, studies have been performed to evaluate the cellulolytic and hemicellulolytic activities of fungi and cellulose-decomposing bacteria isolated from coffee residue compost (CRC) and sawdust compost (SDC) by using azurine cross-linked substrates for cellulase, xylanase, β-glucanase, and mannanase (12, 13).

Phosphorus (P) is the most essential nutritional element for sustaining all life, and hence food production, on our planet (2, 24). This element is also a component of key molecules such as nucleic acids, phospholipids, and ATP. Consequently, plants cannot grow without a reliable supply of P (53). Over the last century, P resources have been intensively depleted by human activities. On the basis of the current rate of depletion, phosphate reservoirs will not last more than 100 years (56). This scarcity of P resources demands effective usage of all allowed P sources, including inorganic and organic P accumulated in waste materials and soil for sustainable agriculture. Compost amendment also enhances biological properties such as phosphate-solubilizing bacteria, microbial biomass P, and improved P availability and utilization by plants in andosols and granitic regosols (62, 63).

Phytate (*myo*-inositol hexakisphosphate) exists in a wide diversity of environments, including arable, forest, and grassland soils, as well as aquatic environments. In the terrestrial ecosystem, phytate is generated by plants (59). In most plants, a large portion of P exists in the form of phytate as a complex salt of calcium or magnesium, mainly in seeds. Phytate P constitutes the major portion (60–82%) of total P in cereal grains, oilseeds, and grain legumes, with 1–25% of total P being found in various roots and tubers and small amounts in leaves and 56–77% of total P in rice bran and various oilseed meals (48). Plants and non-ruminant animals poorly utilize these phytates by direct means (19). For instance, phytate in the soil cannot be utilized directly by plants until it has been hydrolyzed to release phosphate (40). Improving the bioavailability of phytate P to plants might be beneficial for reducing the amount of P fertilization in agricultural environments (30). Phytate has the ability to bind strongly to mineral surfaces, especially clays (7), and forms insoluble metal complexes (8, 21). These properties may render phytate resistant to mineralization by microbial attack (59), resulting in its accumulation in soils.

The degradation of phytate is catalyzed by enzymes called phytases (*myo*-inositol hexakisphosphate phosphohydrolases; EC 3.1.3.8, 3.1.3.26, and 3.1.3.72), which are produced by various organisms, such as plants, animals, and micro-organisms (35). A large number of microorganisms, including bacteria, yeasts, and filamentous fungi, have been identified as phytase producers (29, 31, 44, 49, 61). Phytate-hydrolyzing microorganisms inhabit wide and diverse environments, indicating that the biodegradation of phytate might be accomplished in a variety of ecosystems (23). It has been shown that phytate-utilizing bacteria may improve the acquisition of P by plants (50); however, few studies have focused on the degradation of phytate in organic wastes (16). Composting is an important process that may improve the bioavailability of P through the degradation of organic P by microorganisms (15); therefore, the evaluation of micro-organisms that inhabit composts and their phytate degradation ability might contribute towards improving the quality of composts.

In the current study, we aimed to investigate the phytate-degrading ability of fungi and bacteria isolated from SDC and CRC. Fungal and bacterial isolates were screened to select effective isolates by the plate assay using phytase screening medium (PSM) containing calcium phytate because phyate mainly exists as insoluble salts bound with metals such as calcium and magnesium in organic materials. The phytase activity in liquid culture and the degradation ability of phytate in organic material composed of mushroom media residue and rice bran were evaluated using the selected isolates.

## Materials and Methods

### Compost samples

SDC produced from the residue of culture media for mushrooms was obtained from the Agricultural Cooperative Association of Saitama Prefecture, while CRC was collected from a composting center located in Hiroshima Prefecture. In brief, these materials were produced by composting heaps that were turned periodically for 2–3 months until they reached maturity. Compost samples (0.4 g) were digested with a 1:1 mixture of concentrated H_2_SO_4_ and concentrated HNO_3_ (10 mL) in a Kjeldahl flask for total P analysis. P content was determined spectrophotometrically (UV mini 1240; Shimadzu, Kyoto, Japan) using the ammonium molybdate-ascorbic acid method (43). Total C and total N contents of the composts were determined using a combustion method with a CN analyzer (MT-700; Yanaco Kyoto, Japan). The chemical properties of SDC and CRC are presented in Table 1.

### Fungal and bacterial isolates

The fungal isolates used in this study were obtained from SDC and CRC to evaluate their cellulolytic and hemicellulolytic activities (12). In total, 47 isolates were used, incuding nine *Mucor*, seven *Penicillium*, three *Galactomyces*, 11 *Coniochaeta*, 14 *Trichoderma*/*Hypocrea*, two *Aspergillus*, and one *Fusarium*. The 21 cellulolytic bacterial isolates examined in this study, which were obtained from SDC and CRC (13), were four *Streptomyces*, 13 *Microbispora*, two *Paenibacillus*, and two *Cohnella*.

The bacteria that formed clear zones around their colonies were isolated from the same SDC and CRC using the dilution plate method. The primary suspensions were prepared by suspending an equivalent of 10 g oven-dry weight of each compost type in 90 mL sterilized distilled water and shaking (150 rpm) for 30 min at room temperature. Then, 10-fold serial dilutions were prepared in sterile distilled water. One hundred microliters from the 10^−4^ dilution of each compost type was spread on phytase screening medium (PSM), according to the method used by Yoon *et al.* (65). PSM agar is composed of calcium phytate, 5.0 g L^−1^; (NH_4_)_2_SO_4_, 3.0 g L^−1^; CaCl_2_, 0.1 g L^−1^; MnSO_4_·5H_2_O, 0.1 g L^−1^; FeSO_4_·7H_2_O, 0.1 g L^−1^; and glucose, 10 g L^−1^. The pH was adjusted to 7.0. The plates were incubated at 35°C for 7 d because of the better formation of clear zones at 35°C than 30°C. Five plates were prepared for each compost type. The colonies surrounded by large clear zones were selected from the most typical plate and transferred to trypto-soya agar (TSA; Nissui Pharmaceutical, Tokyo, Japan) plates. A single colony of each isolate was frequently transferred on TSA to obtain pure cultures. Four bacteria were isolated from the SDC and two were isolated from the CRC.

### DNA extraction and polymerase chain reaction amplification

DNA extraction and polymerase chain reaction (PCR) amplification from the bacterial isolates were performed according to the methods used in a previous study (13). In brief, genomic DNA was extracted using the boiling method. DNA solution was used as a template for PCR amplification. PCR was performed with universal bacterial primers for the 16S rRNA gene; 27F and 1492R were used as the forward and reverse primers, respectively (37). PCR amplifications were conducted using the GeneAmp PCR system 2700 by following the conditions described in a previous study (13). PCR products were analyzed by agarose gel electrophoresis and then purified using the QIAquick PCR purification kit (Qiagen, Hilden, Germany), according to the manufacturer’s instructions.

### Sequencing and data analysis

The 16S rRNA gene sequences of the isolated bacteria were determined by direct sequencing of the purified PCR-amplified 16S rDNA fragments. The amplified 16S rRNA fragments were sequenced using the BigDye Terminator v3.1 Cycle Sequencing Kit (Applied Biosystems, Foster City, CA, USA) on an Applied Biosystems 3730*xl* DNA analyzer. An almost complete 16S rRNA gene was sequenced from each isolate using the following oligonucleotides: 27F, 515F (60), F984 (22), and 519R (37). The 16S rDNA sequences of the isolated bacteria were compared with those of closely related species deposited in the GenBank database (http://blast.ncbi.nlm.nih.gov/Blast.cgi) using the nucleotide-nucleotide basic local alignment search tool (BLASTn). A phylogenetic tree based on 16S rRNA gene partial sequences was constructed using the neighbor-joining method on the MEGA5 program (55).

### Screening of fungal and bacterial isolates by the plate assay

Fungal isolates were grown on potato dextrose agar (PDA; Nissui Pharmaceutical, Tokyo, Japan) and then agar plugs (6 mm in diameter), with mycelium excised from the plate of each isolate. The agar plugs were placed in the center of a PSM agar plate surface in three replicates and incubated at 25°C for 7 d. In the case of bacteria, bacterial cells grown on TSA were inoculated at a point on the PSM agar plate surface and incubated for 7 d at 35°C. After incubation, the diameter of the clear zone and diameter of fungal and bacterial growth were measured. Three replicates of each experiment were conducted. Screening was carried out comparing the clear zone diameter and the ratio of clear zone diameter to growth diameter (CD/GD ratio), since the growth of microbes greatly affects the size of the clear zone.

### Measurement of phytase activity

The selected eight fungal isolates (SDCF1, SDCF3, SDCF5, SDCF11, SDCF17, SDCF18, SDCF22, and CRCF1) and four bacterial isolates (PSDCB1, PSDCB3, PCRCB18, and PCRCB19) were inoculated at a rate of 10^5^ and 10^6^ cfu mL^−1^, respectively, into 30 mL PSM broth (65) with modification, using sodium phytate instead of calcium phytate at the same rate (5.0 g L^−1^) in 100 mL Erlenmeyer flasks. Sodium phytate was added separately to autoclaved media containing other constituents after pH adjustment and filtration with a sterile membrane filter (DISMIC 25CS020AS, 0.2 μm; Toyo Roshi, Tokyo, Japan). The pH of PSM broth was adjusted to 5.5 for fungi and to 7.0 for bacteria. The media was incubated with orbital shaking (120 rpm) at 30°C for 5 d. All treatments were conducted in triplicate. After incubation, the liquid cultures were centrifuged at 3,000 rpm for 15 min and then filtered through a sterile membrane filter (DISMIC 25CS020AS, 0.2 μm). Phytase activity of the culture filtrates was measured according to the method described by George *et al.* (18) in larger volume. Briefly, the reaction solution composed of 3 mL of 25 mM MES buffer (pH 5.5), 1 mL of 10 mM sodium phytate (pH adjusted to 5.5) as the substrate and 1 mL culture filtrate was incubated at 37°C for 1 h. The reaction was stopped by adding 5 mL of 10% trichloroacetic acid. The liberated inorganic P in the solution was determined colorimetrically using a malachite green reagent (26), and phytase activity was expressed as μmol inorganic P released per 1 mL culture filtrate within a minute. Inorganic P in the culture filtrate was also determined using the malachite green reagent.

### Incubation of organic materials inoculated with the selected bacteria and fungi

Organic materials inoculated with selected microorganisms were cultured in a semisolid state, according to the method published by Lopez *et al.* (38), to investigate their ability to degrade phytate in organic materials. A mixture of 3.49 g (2.0 g in dry weight) mushroom media residue, which was mainly composed of sawdust and 0.53 g (0.5 g in dry weight) rice bran, was placed in a 100 mL Erlenmeyer flask and 18 mL distilled water was added. The mixture of organic materials was inoculated with 0.5 mL fungal or bacterial suspensions (10^6^ cfu g^−1^ dry matter) after autoclaving at 121°C for 20 min and incubated with rotary shaking (120 rpm) at 30°C for 7 and 14 d in darkness. After incubation, the culture was autoclaved at 121°C for 20 min, dried at 80°C for 12 h, and weighed. Three replicates of all treatments were performed. The chemical properties of mushroom media residue and rice bran are presented in Table 2. The pH of the mixture of mushroom media residue and rice bran was 6.0 (1:10, H_2_O).

### Determination of phytate and inorganic P

Phytate P was measured according to the method used by Raboy *et al.* (47), with some modifications. Briefly, 30 mL of 0.4 M HCl:0.7 M Na_2_SO_4_ was added to each 100 mL Erlenmeyer flask containing whole dried organic materials and extracted by shaking (120 rpm) at room temperature for 12 h after sealing. Three replicates of all treatments were performed. The suspension was centrifuged at 3000 rpm for 15 min and filtered. Ten milliliters of distilled water and 5 mL of 15 mM FeCl_3_:0.2 M HCl were added to 10 mL filtrate in a 50 mL glass centrifuge tube. The mixture was heated in a boiling water bath for 30 min, allowed to cool down, and centrifuged at 3000 rpm for 15 min, followed by washing twice with 0.2 M HCl to obtain the ferric phytate precipitate. The precipitated ferric phytate was digested with 10 mL H_2_SO_4_:HNO_3_ (1:1, v/v). Phytate P in the diluted solution after digestion and inorganic P (orthophosphate P) in the filtrate after extraction with 0.4 M HCl:0.7 M Na_2_SO_4_ were determined colorimetrically (43).

### Statistical analysis

Statistical analyses were performed using JMP 8 (SAS Institute). Pair-wise correlations were calculated between clear zone diameters and the CD/GD ratio in the plate assay that contained fungal isolates and phytate P and inorganic P contents in organic materials inoculated with fungal isolates after incubation.

### Accession numbers of nucleotide sequences

Nucleotide sequences of the partial 16S rRNA genes of the bacterial isolates were deposited in GenBank under accession numbers JQ864387–JQ864392.

## Results

### Isolation and phylogenetic analysis of bacterial isolates

**Fig. 1:**
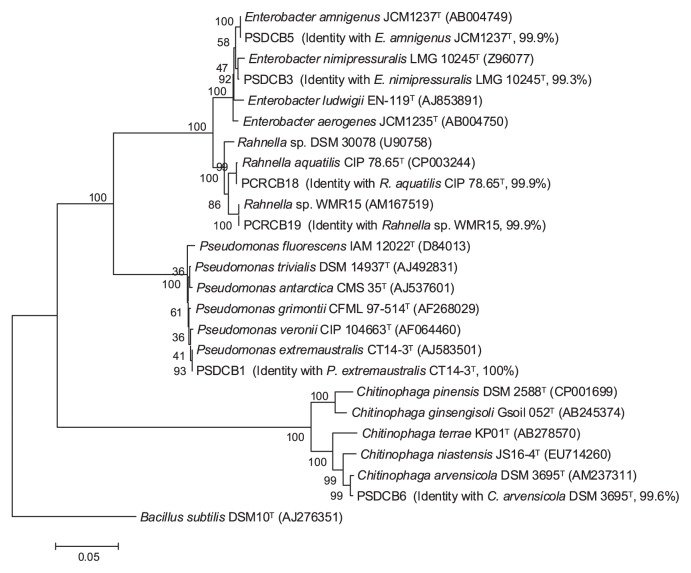
Phylogenetic tree based on the relationship among the 16S rRNA sequences of phytate-degrading bacteria isolated from SDC and CRC and type strains of close species. *Rahnella* sp. WMR15 and *Rahnella* sp. DSM 30078 were chosen as the representatives of *Rahnella* genomospecies 2 and genomospecies 3. Bootstrap values of neighbor-joining analysis of 1,000 replications are shown on the branches as percentages. Scale bar represents the number of substitution per nucleotide position.

Four isolates (PSDCB1, PSDCB3, PSDCB5, and PSDCB6) were isolated from SDC and two isolates (PCRCB18 and PCRCB19) from CRC, and they were subjected to phylogenetic analysis and a phytate degradation study. The results of the BLASTn search (Table 3) and the constructed phylogenetic tree (Fig. 1) based on the sequences of 16S rRNA genes revealed that the isolated bacteria belonged to four genera (*Pseudomonas*, *Enterobacter*, *Chitinophaga*, and *Rahnella*). Genera *Pseudomonas*, *Enterobacter*, and *Chitinophaga* were isolated from SDC, while the *Rahnella* genus was isolated from CRC. The isolate PSDCB1 was closest to *P. extremaustralis* CT14-3^T^ in the phylogenetic tree, with 100% similarity. The sequences of PSDCB3 and PSDCB5 were matched with *E. nimipressuralis* LMG 10245^T^ and *E. amnigenus* JCM1237^T^, with 99.3 and 99.9% similarity, respectively. The identity of isolate PSDCB6 with the closest type strain *C. arvensicola* DSM 3695^T^ was 99.6%. Both bacteria isolated from CRC belonged to the *Rahnella* genus, as revealed by the phylogenetic tree and BLASTn results. Isolate PCRCB18 showed 99.9% similarity to *R. aquatilis* CIP 78.65^T^, while PCRCB19 isolate was closely related to *Rahnella* sp. WMR15 (99.9% identity).

### Screening of bacterial and fungal isolates

The formation of clear zones by fungi (12), cellulolytic bacteria (13), and bacteria isolated from SDC and CRC in this study were investigated on PSM agar plates. The cellulolytic bacteria did not produce clear zones around their colonies (data not shown). All the fungi from SDC, except the SDCF16 isolate, exhibited clear zone formation (Table 4). The fungi isolated from SDC belonged to genera *Mucor* (nine isolates), *Penicillium* (five isolates), and *Galactomyces* (three isolates). The fungi from CRC belonged to genera *Coniochaeta* (eight isolates), *Fusarium* (one isolate), *Penicillium* (one isolate), and *Aspergillus* (one isolate).

**Fig. 2:**
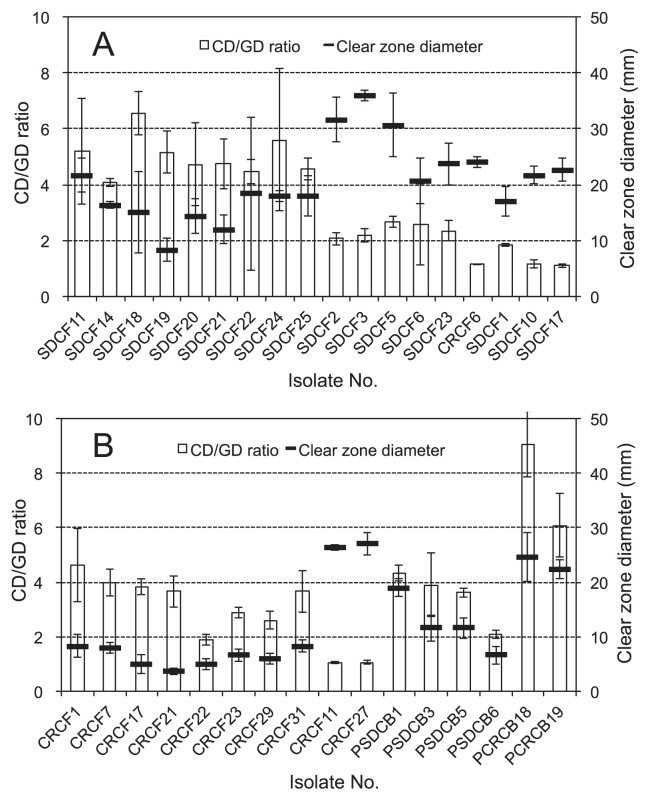
Clear zone diameter and CD/GD ratio of fungi and bacteria isolated from SDC and CRC in the plate assay. CD/GD ratio is the ratio of the clear zone diameter to growth diameter. Clear zone diameters around the colonies are presented in mm. Vertical bars indicate the standard deviations. Panel A shows results of *Mucor* sp. SDCF11, 14, 18, 19, 20, 21, 22, 24 and 25, *Penicillium* sp. SDCF2, 3, 5, 6, 23 and CRCF6, and *Galactomyces* sp. SDCF1, 10 and 17. Panel B shows the results of *Coniochaeta* sp. CRCF1, 7, 17, 21, 22, 23, 29 and 31, *Aspergillus* sp. CRCF11, *Fusarium* sp. CRCF27, *Pseudomonas* sp. PSDCB1, *Enterobacter* sp. PSDCB3 and 5, *Chitinophaga* sp. PSDCB6, and *Rahnella* sp. PCRCB18 and 19.

Among all the fungal isolates from SDC and CRC, the *Penicillium* isolates showed the greatest clear zone diameters (SDCF3, followed by SDCF2 and SDCF5), while the largest clear zone was formed by the *Mucor* isolates (SDCF18, followed by SDCF 24 and SDCF11) (Fig. 2). In the isolates from CRC, the highest CD/GD ratio was displayed by CRCF1 (*Coniochaeta* sp.), while CRCF27 (*Fusarium* sp.), CRCF11 (*Aspergillus* sp.), and CRCF6 (*Penicillium* sp.) formed greater clear zones than CRCF1. The isolates closest to *Rahnella* sp. (PCRCB18 and PCRCB19) showed higher CD/GD ratios and clear zone diameters than all other isolates (Fig. 2). Isolate PCRCB18 showed the highest ability to form clear zone. Among isolates from SDC, PSCDB1 (*Pseudomonas* sp.) revealed the highest CD/GD ratio and clear zone diameter, followed by PSCDB3 and PSCDB5 (*Enterobacter* sp.) at similar levels.

Phytate-degrading activity was subsequently evaluated for the most effective eight fungal isolates (SDCF1, SDCF3, SDCF5, SDCF11, SDCF17, SDCF18, SDCF22, and CRCF1) in the genera *Mucor*, *Penicillium*, *Galactomyces*, and *Coniochaeta* and the most effective four bacterial isolates (PSDCB1, PSDCB3, PCRCB18, and PCRCB19), which were selected on the basis of their higher CD/GD ratio or greater clear zone diameters. Among *Mucor* isolates, SDCF11 showed the highest clear zone diameter, SDCF18 showed the highest CD/GD ratio, and SDCF22 displayed relatively high CD/GD ratio and clear zone diameter. Isolate SDCF3 was selected because of its highest clear zone diameter, and SDCF5 was selected because it had the highest CD/GD ratio among the *Penicillium* isolates. Two *Galactomyces* isolates (SDCF1 and SDCF17) were selected for the same reason. *Coniochaeta* isolate CRCF1 showed the highest CD/GD ratio and clear zone diameter among the *Coniochaeta* isolates. Four bacterial isolates (PSDCB1, PSDCB3, PCRCB18, and PCRCB19) showed higher CD/GD ratios and the greatest clear zone diameters.

### Phytase activity of the selected fungi and bacteria

**Fig. 3:**
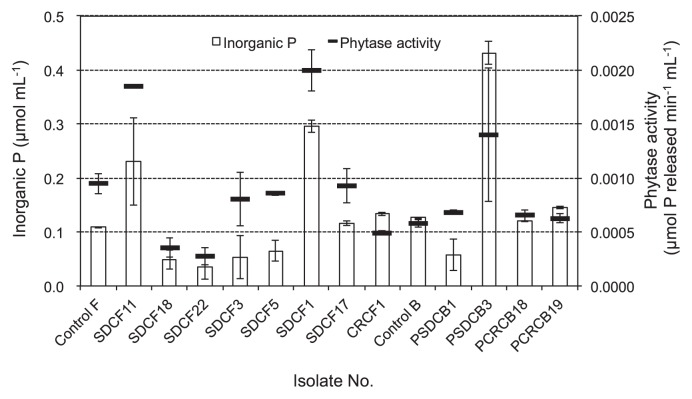
Inorganic P concentrations and phyatase activity in the culture filtrates of selected fungal and bacterial isolates grown in liquid media containing sodium phytate as sole P source. Vertical bars indicate the standard deviations. Legend: Control F, no fungal inoculation; Control B, no bacterial inoculation; SDCF11, 18 and 22, *Mucor* sp.; SDCF3 and 5, *Penicillium* sp.; SDCF1 and 17, *Galactomyces* sp.; CRCF1, *Coniochaeta* sp; PSDCB1, *Pseudomonas* sp.; PSDCB3, *Enterobacter* sp.; PSDCB18 and 19, *Rahnella* sp.

Isolate SDCF1 (*Galactomyces* sp.) and SDCF11 (*Mucor* sp.) increased the concentrations of inorganic P (0.30 and 0.23 μmol P mL^−1^, respectively) more than other tested fungal isolates (Fig. 3). On the other hand, some isolates such as *Penicillium* isolates (SDCF3 and SDCF5) and *Mucor* isolates (SDCF18 and SDCF22) reduced the level of inorganic P in the culture medium compared to the control (medium without inoculation) after incubation. Inorganic P in the culture of SDCF17 (*Mucor* sp.) and CRCF1 (*Coniochaeta* sp.) increased slightly. In bacterial isolates PSDCB3 (*Enterobacter* sp.) showed the highest inorganic P in the culture filtrate, while PSDCB1 (*Pseudomonas* sp.) decreased the inorganic P concentration in the culture filtrate. *Rahnella* isolates (PCRCF18 and PCRCF19) exhibited similar levels of inorganic P concentrations to the control. Phytase activity in the culture filtrate of fungi and bacteria showed a similar tendency to the concentrations of inorganic P released from sodium phytate (Fig. 3). The highest phytase activity (0.0020 μmol P released min^−1^ mL^−1^) in fungal culture was revealed by SDCF1 (*Galactomyces* sp.) followed by SDCF11 (*Mucor* sp.) (0.0019 μmol P released min^−1^ mL^−1^). Meanwhile SDCF17 (*Galactomyces* sp.), SDCF3 and SDCF5 (*Penicillium* sp.) showed nearly equal levels of phytase activity to the control, and the activity of *Mucor* isolates (SDCF18 and SDCF 22) and *Coniochaeta* isolate (CRCF1) was low. The bacterial isolate PSDCB3 (*Enterobacter* sp.) displayed higher phytase activity (0.0014 μmol P released min^−1^ mL^−1^) than the other bacterial isolates. Other bacterial isolates, PSDCB1 (*Pseudomonas* sp.), PSDCB18, and PCRCB19 (*Rahnella* sp.), showed activity at the similar level to the control.

### Degradation of phytate in organic materials inoculated with the selected fungi and bacteria

**Fig. 4:**
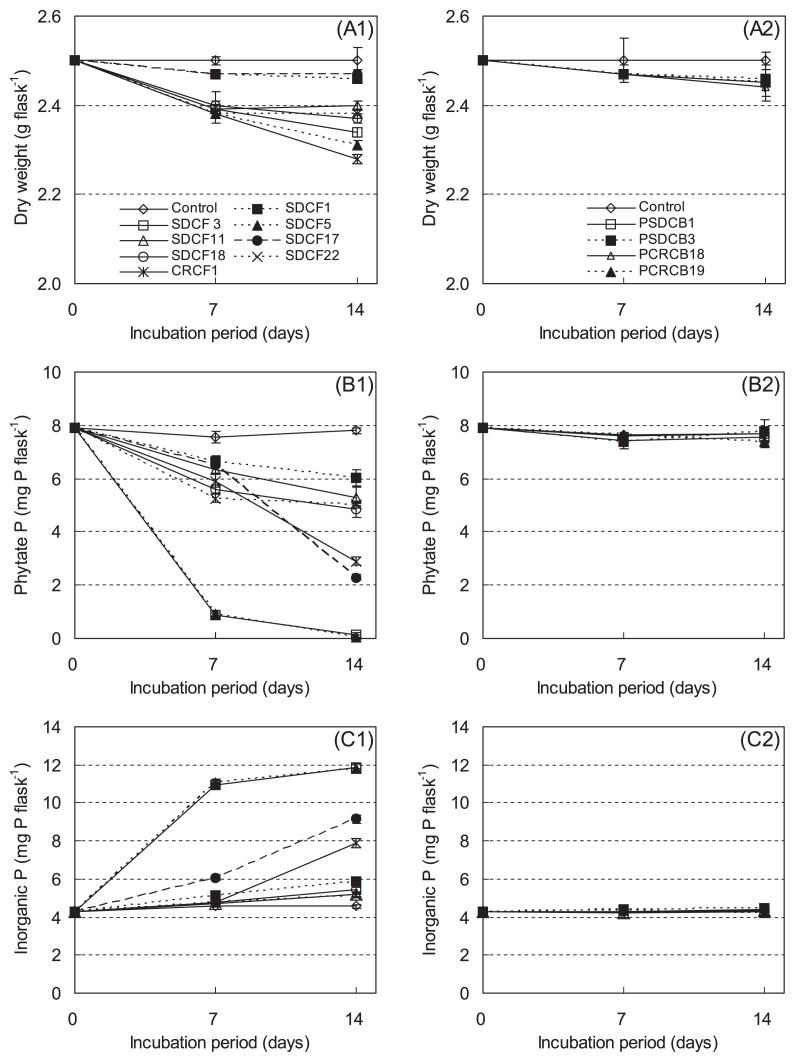
Changes in dry weight, phytate P and inorganic P contents of organic materials inoculated with isolates of fungi (A1, B1 and, C1) and bacteria (A2, B2 and, C2) during incubation. Vertical bars indicate the standard deviations. SDCF1 and 17, *Galactomyces* sp.; SDCF3 and 5, *Penicillium* sp.; SDCF11, 18 and 22, *Mucor* sp.; CRCF1, *Coniochaeta* sp.; PSDCB1, *Pseudomonas* sp.; PSDCB3, *Enterobacter* sp.; PSDCB18 and 19, *Rahnella* sp.

During incubation with selected fungal isolates, the phytate P contents of the organic materials were reduced in comparison with the control treatment, as illustrated in Fig. 4. The most active isolates concerning the degradation of phytate were *Penicillium* isolates (SDCF3 and SDCF5). The phytate P levels of organic materials inoculated by SDCF3 and SDCF5 were reduced by 88.3 and 87.8%, respectively, after 7 d and by 98.4 and 99.2%, respectively, after 14 d. The amounts of decreased phytate P were 6.68 and 6.64 mg flask^−1^ after 7 d and 7.68 and 7.74 mg flask^−1^ after 14 d, as compared with the amount of phytate P in the control. *Galactomyces* sp. SDCF1 exhibited the lowest decomposing rate, where phytate P was reduced by 23.0% over a 14-d period; however, another *Galactomyces* sp., SDCF17, reduced phytate P by 13.9% and 71.6%, in comparison with that in the control after 7 and 14 d of incubation, respectively. *Coniochaeta* sp. CRCF1 exhibited relatively high phytate degradation ability, which mineralized 21.9 and 63.0% phytate P after 7 and 14 d, respectively. The phytate degradation ability of *Mucor* isolates (SDCF11, SDCF18, and SDCF22) was relatively low, since they reduced phytate P by 34.7, 37.9, and 35.3%, respectively, after 14 d. The dry weights of organic materials were reduced differently by the growth of fungal isolates. The amount of dry weight loss by fungal isolates was similar at 4.0–4.8% after 7 d of incubation, except for *Galactomyces* isolates (SDCF1 and SDCF17); however, *Coniochaeta* sp. CRCF1 and *Penicillium* sp. SDCF5 showed the greatest decrease in dry weight of organic materials (8.8 and 7.6%, respectively) after 14 d. The decrease in dry weight by 2 *Galactomyces* isolates (SDCF1 and SDCF17) was lowest, at 1.6 and 1.2%, respectively, after 14 d (Fig. 4).

The concentrations of inorganic P were increased by inoculation with fungal isolates (Fig. 4), which reflected the reduction of phytate P contents. *Penicillium* isolates (SDCF3 and SDCF5) displayed the highest inorganic P (11.9 and 11.8 mg P flask^−1^, respectively) compared to the control (4.6 mg P flask^−1^) after 14 d of incubation. The inorganic P levels of isolates SDCF3 and SDCF5 (*Penicillium* sp.) sharply increased until 7 d after treatment. In comparison, *Galactomyces* sp. SDCF17 and *Coniochaeta* sp. CRCF1 showed low efficiency in the degradation of phytate during the first week of incubation and effectively increased inorganic P over the second week. The other isolates, including *Mucor* and *Galactomyces* spp., did not show a noticeable increase in inorganic P content during incubation.

The bacterial isolates incubated with organic materials scarcely decreased the dry weights of the organic materials and did not decrease phytate P or increase inorganic P contents in organic materials (Fig. 4).

## Discussion

Isolates PSDCB1, PSDCB3, PSDCB5, PSDCB6, PCRCB18, and PCRCB19 were closest to *Pseudomonas extremaustralis*, *Enterobacter nimipressuralis*, *Enterobacter amnigenus*, *Chitinophaga arvensicola*, *Rahnella aquatilis*, and *Rahnella* sp. WMR15, respectively (Fig. 1). Although *R. aquatilis* is the only species belonging to the genus of *Rahnella*, three genome species of the genus of *Rahnella*, such as *R. aquatilis*, *Rahnella* genomospecies 2, and *Rahnella* genomospecies 3, which cannot be phenotypically differentiated, have been previously reported (6, 52). Isolate PCRCB19 was closest to *Rahnella* sp. WMR15 (52), which is a representative of *Rahnella* genomospecies 2, based on phylogenetic analysis. The bacteria species in these four genera have been widely found in composts. Several species of *Enterobacter* and *Pseudomonas* have been isolated from the active phase of compost (4, 10). In addition, Manjunathan and Kaviyarasan (41) found that bacteria members of the genera *Pseudomonas* and *Enterobacter* were dominant during the composting of sawdust and wheat bran. *Rahnella* sp. was detected during the degradation of the mixture of sewage sludge and food waste (27), and during aerobic degradation of dairy cattle dung (16). *C. arvensicola* was isolated from acidic *Sphagnum* peatlands (45), and *C. eiseniae* was isolated from vermicompost (64).

Phytase activity by various bacteria, including genera such as *Bacillus*, *Citobacter*, *Escherichia*, *Enterobacter*, *Klebsiella*, and *Pseudomonas*, has been subjected to review (31). The phytate-utilizing ability of several bacterial isolates during the aerobic degradation of dairy cattle dung, which belong to genera *Bacillus*, *Enterobacter*, *Escherichia*, *Shigella*, *Streptomyces*, *Rahnella*, and *Ochrobactrum*, has been reported using the PSM plate assay (16); however, degradation of phytate by *Chitinophaga* sp. has not been reported.

The fungal isolates that exhibited clear zone formation in the plate assay belonged to genera *Mucor*, *Penicillium*, *Galactomyces*, *Coniochaeta*, *Fusarium*, and *Aspergillus* (Table 4). *Mucor* spp. have been commonly reported for phytate degradation (3, 51). For instance, Howson and Davis (25) described the phytase activities of several fungal genera including *Aspergillus*, *Mucor*, and *Geotrichum* (teleomorph: *Galactomyces*) (9). The production of phytase by *Penicillium* and *Fusarium* has also been documented (42, 57); however, reports of phytate degradation by *Coniochaeta* sp. could not be found. Although the phytase activity of *Trichoderma* spp., such as *T. harzianum* and *T. viride*, was indicated by Aseri *et al.* (1), isolates belonging to the *Trichoderma* genus did not exhibit any ability to form a clear zone around their colonies in this study.

Among all the tested fungi from SDC and CRC, *Penicillium* sp. (SDCF3, followed by SDCF2 and SDCF5) showed the greatest clear zones, while the highest CD/GD ratio was revealed by *Mucor* sp. (SDCF18, SDCF 24, and SDCF11) (Fig. 2). The ability to form a clear zone by isolates from CRC was lower than from SDC. Among the isolates from CRC, the highest CD/GD ratio was observed in CRCF1 (*Coniochaeta* sp.). Isolates CRCF27 (*Fusarium* sp.), CRCF11 (*Aspergillus* sp.), and CRCF6 (*Penicillium* sp.) formed greater clear zones than CRCF1. *Rahnella* spp. (PCRCB18 and PCRCB19) created a higher CD/GD ratio and larger clear zones than other bacteria (Fig. 2), while PCRCB18 showed the largest clear zone. PSCDB1 (*Pseudomonas* sp.), followed by PSCDB3 and PSCDB5 (*Enterobacter* sp.), had moderate ability. No cellulolytic bacteria isolated from SDC and CRC (13), which belong to the genera of *Streptomyces*, *Microbispora*, *Paenibacillus*, and *Cohnella*, formed clear zones around their colonies in the plate assay (data no shown). There has been no report regarding the production of phytase by genera *Microbispora* and *Cohnella*. López-López *et al.* (39) identified *Streptomyces kunmingensis* as one of the phytate-degrading bacteria; however, isolates of *Streptomyces* sp. from SDC and CRC in the current study did not form clear zones. The two *Paenibacillus* isolates also did not form clear zones, although a phytase-like protein of *Paenibacillus* was encoded (32). These variations may be caused by differences among identified species.

The highest phytase activity in liquid medium was revealed by SDCF1 (*Galactomyces* sp.) and SDCF11 (*Mucor* sp.) (Fig. 3). The increases in the concentrations of inorganic P in the culture filtrates revealed by *Galactomyces* and *Mucor* isolates were owing to the higher phytase activity. Some isolates belonging to genera *Mucor* and *Penicillium* reduced inorganic P content in the liquid medium. This result may be caused by the uptake of inorganic P in the growth of fungal isolates. Phytase activity and inorganic P in culture filtrates of liquid media inoculated with selected fungal and bacterial isolates did not show a relationship with their CD/GD ratio and clear zone diameters.

The highest degrading ability of phytate in organic materials among fungi was revealed by *Penicillium* isolates SDCF3 and SDCF5 (Fig. 4), which might be due to their larger production of phytase. In addition, their greater growth levels might have been supported by cellulolytic and hemicellulolytic abilities, such as cellulase, xylanase, β-glucanase, and mannanase activities (12), as shown by the higher decrease of dry weight. In contrast, *Galactomyces* isolates, which did not show any cellulolytic or hemicellulolytic activities (12), showed the lowest loss of dry weight; however, *Galactomyces* isolate SDCF17 degraded phytate in large quantities (after that of *Penicillium* isolates) after 14 d of incubation. Meanwhile, *Galactomyces* isolate SDCF1 decomposed the smallest amount of phytate among all tested fungi. Phytase production might be related to a combination of growth, different species, and strains. Although a higher production of phytase by *Mucor* spp. such as *M. racemosus* and *M. hiemalis* has been reported (3, 5, 51), *Mucor* isolates (SDCF11, SDCF18, and SDCF22), which were closest to *M. circinelloides* (12), revealed relatively low phytate degradation ability in this study, and a moderate reduction of dry weight. This difference may be caused by different species. *Mucor* isolates (SDCF11, SDCF18, and SDCF22) showed β-glucanase and mannanase activities, but not cellulase or xylanase activities (12). In addition, it should be mentioned that the difference in decreased phytate P (2.50, 2.95, and 2.76 mg P flask^−1^) and increased inorganic P (0.65, 0.86, and 0.56 mg P flask^−1^) levels in the organic materials inoculated with *Mucor* isolates (SDCF11, SDCF18, and SDCF22) were markedly larger than other fungal isolates. James and Casida (28) reported that *M. racemosus* could accumulate 5–6% phosphorus in its dry mycelium, and approximately 58% phosphorus in the mycelium existed as inorganic polyphosphate. *Coniochaeta* isolate CRCF1 showed higher degradation ability than *Mucor* isolates, and the largest loss in dry weight in the incubation experiment, even though reports about phytate degradation by *Coniochaeta* could not be found. *Coniochaeta* isolate CRCF1 exhibited xylanase, β-glucanase, and mannanase activities (12). The cellulolytic and hemicellulolytic abilities of fungi could help their growth in organic materials. The degradation ability of phytate in organic materials inoculated with selected fungi, however, did not show the relationship with their phytase activities in liquid culture. The measurement of phytase activity using liquid culture should be further investigated.

Previous studies have also reported that phytase production is affected by the components of the media, including the concentration of inorganic P and phytate in the media (3, 11, 14, 17, 20, 26, 34, 36, 46, 54). The effects of inorganic P and phytate concentration on the induction of phytase production vary among microorganisms. Fredrikson *et al.* (14) reported that calcium phytate in the PSM was hydrolyzed to inorganic orthophosphate by autoclaving. The highest phytate degradation by *Rhizopus microsporus*, *Aspergillus ficuum*, and *Geotrichum candidum* was observed using synthetic liquid medium containing both inorganic P and phytate, which indicates that inorganic P might facilitate initial microbial growth and an increase of phytase activity following the decrease of inorganic P (14). In comparison, phytase synthesis by fungi decreased markedly when increasing amounts of phosphate were added to the medium, while the growth of microbes increased with increased phosphate (3, 11, 17, 25, 46, 54); however, the concentrations of inorganic P that limited the induction of phytase differed among species and strains. A similar phenomenon was observed for bacteria, such as *Bacillus laevolacticus* (20) and *Mitsuokella jalaludinii* (36). In the case of *B. subtilis* VTT E-68013, the medium containing inorganic phosphate and phytate did not induce phytase production; however, synthetic medium containing phytate as the sole P source could induce it, indicating that the synthesis of phytase might be regulated by a combination of phytate and limiting inorganic P (34).

The ability of fungal isolates to degrade phytate was evaluated using the clear zone diameter and CD/GD ratio, because their growth obviously affected the clear zone diameter; however, the CD/GD ratio was not significantly correlated with the clear zone diameter (R=−0.5058; *P*=0.2009), phytate P (R=0.2472; *P*=0.5550, R=0.4516; *P*=0.2614), or inorganic P (R=−0.4937; *P*=0.2137, R=− 0.5968; *P*=0.1183). The significantly negative correlation between the clear zone diameter and phytate P after 7 d, and the significantly positive correlations between the clear zone diameter and inorganic P after 7 and 14 d demonstrated that the growth of fungi might have a major effect on the degradation of phytate in organic materials, as well as phytase-producing ability, although other types of organic materials were not tested in this study (Table 5). Furthermore, there were significantly negative correlations between phytate P and inorganic P after incubation. Hence, the clear zone diameter in the PSM plate assay might be a more useful method to estimate the ability to degrade phytate in fungal studies, rather than the CD/GD ratio.

Bacterial isolates that were applied in the incubation experiment did not display any significant activity on phytate degradation of the organic materials (Fig. 4), although the bacterial isolate PSDCB3 (*Enterobacter* sp.) showed phytase activity in liquid culture. The lack of a useable carbon source for bacterial isolates may cause less growth, while sufficient amounts of inorganic P in organic materials may affect phytase production. The phytase activity of bacterial isolates other than PSDCB3 should be confirmed under different conditions.

In conclusion, fungi probably play a major role in phytate degradation during the composting process since fungal isolates selected by the PSM plate assay degraded phytate in organic materials, but selected bacterial isolates did not. Two *Penicillium* sp., SDCF3 and SDCF5, showed the highest degradation ability of phytate, and *Coniochaeta* isolate CRCF1 exhibited the highest degradation ability of organic materials and relatively high degradation ability of phytate, suggesting their considerable role during composting. This is the first report about phytate degradation by *Coniochaeta* sp.

## Figures and Tables

**Table 1:** Chemical properties of SDC and CRC

Compost used^a^	Dry matter (g kg^−1^)	C	N	P	C/N ratio

		(g kg^−1^)^b^			
SDC	538	456	14.7	12.3	30.0
CRC	390	448	21.4	0.7	20.9

a SDC, sawdust compost; CRC, coffee residue compost.

b Values are expressed on an oven-dry basis.

**Table 2:** Chemical properties of organic materials

Organic material	Dry matter (g kg^−1^)	C	N	P	Phy-P^a^	IP^b^	C/N ratio	pH (1:10, H_2_O)
	
		(g kg^−1^)^c^			(g kg^−1^)^c^			
Mushroom media residue	573	447	11.2	2.2	ND^d^	1.3	39.9	5.6
Rice bran	935	457	22.2	19.1	16.9	2.0	20.6	6.9

a Phy-P, Phytate P.

b IP, Inorganic P (extracted with 0.4 M HCl:0.7 M Na_2_SO_4_).

c Values are expressed on an oven-dry basis.

d ND, not detectable.

**Table 3:** Closest relatives of bacterial isolates from SDC and CRC in the BLASTn results for 16S rRNA gene sequences

Isolate No.	Accession No.	Closest relative (Accession No.)^a^	Length	Identity (%)
PSDCB1	JQ864387	*Pseudomonas extremaustralis* (AJ583501)	1459	1459/1459 (100)
PSDCB3	JQ864388	*Enterobacter* sp. (DQ279307)	1462	1455/1457 (99.9)
PSDCB5	JQ864389	*Enterobacter* sp. (JF939050)	1462	1461/1462 (99.9)
PSDCB6	JQ864390	*Chitinophaga arvensicola* (AB681051)	1449	1444/1450 (99.6)
PCRCB18	JQ864391	*Rahnella aquatilis* (CP003244)	1465	1463/1465 (99.9)
PCRCB19	JQ864392	*Rahnella* sp. (AM167519)	1465	1464/1465 (99.9)

a Closest relative was chosen from highly ranked representative isolates in the BLASTn results.

**Table 4:** List of fungal isolates that formed or did not form clear zones around their colonies in the plate assay

Genus	Clear zone formed				Clear zone not formed			
	
	Isolate No.				Isolate No.			
*Mucor*	**SDCF11**	SDCF14	**SDCF18**	SDCF19				
	SDCF20	SDCF21	**SDCF22**	SDCF24				
	SDCF25							
*Penicillium*	SDCF2	**SDCF3**	**SDCF5**	SDCF6	CRCF12			
	SDCF23	CRCF6						
*Galactomyces*	**SDCF1**	SDCF10	**SDCF17**					
*Coniochaeta*	**CRCF1**	CRCF7	CRCF17	CRCF21	CRCF20	CRCF24	CRCF30	
	CRCF22	CRCF23	CRCF29	CRCF31				
*Trichoderma/Hypocrea*					CRCF2	CRCF3	CRCF8	CRCF9
					CRCF10	CRCF13	CRCF14	CRCF15
					CRCF16	CRCF18	CRCF28	CRCF4
					CRCF5	CRCF26		
*Aspergillus*	CRCF11				SDCF16			
*Fusarium*	CRCF27							

**Table 5:** Correlation coefficients between clear zone diameters and the ratio of clear zone diameter to growth diameter (CD/GD ratio) in the plate assay with fungal isolates, phytase activity and inorganic P in culture filtrate, and phytate P and inorganic P contents in organic materials inoculated with fungal isolates after incubation

	Clear zone diameter	CD/GD ratio	Phytate P (7 d)	Phytate P (14 d)	Inorganic P (7 d)	Inorganic P (14 d)
Clear zone diameter	1					
CD/GD ratio	−0.5058 (*P*=0.2009)	1				
Phytate P (7 d)	−0.8022 (*P*=0.0166)*	0.2472 (*P*=0.5550)	1			
Phytate P (14 d)	−0.6854 (*P*=0.0606)	0.4516 (*P*=0.2614)	0.8278 (*P*=0.0112)*	1		
Inorganic P (7 d)	0.8770 (*P*=0.0042)*	−0.4937 (*P*=0.2137)	−0.9513 (*P*=0.0003)*	−0.8858 (*P*=0.0034)*	1	
Inorganic P (14 d)	0.7273 (*P*=0.0409)*	−0.5968 (*P*=0.1183)	−0.8161 (*P*=0.0135)*	−0.9771 (*P*<0.0001)*	0.9187 (*P*=0.0013)*	1

* Significant correlation at *P*<0.05.
